# Patientenorientierung

**DOI:** 10.1007/s00104-022-01629-4

**Published:** 2022-04-07

**Authors:** André L. Mihaljevic, Christoph Michalski, Udo Kaisers, Guido Strunk

**Affiliations:** 1 Klinik für Allgemein- und Viszeralchirurgie, Universitätsklinikum Ulm, Albert-Einstein-Allee 23, 89081 Ulm, Deutschland; 2 Executive Academy, Wirtschaftsuniversität Wien, Welthandelsplatz 1, 1020 Wien, Österreich; 3 Complexity-Research, Schönbrunner Str. 32/3/20, 1050 Wien, Österreich

**Keywords:** Patientenorientierung, Patientenzentrierung, Patient-reported experience measures, Patient-reported outcome measures, Patient-centeredness, Patient-reported experience measures, Patient-reported outcome measures, Patient outcome assessment

## Abstract

**Hintergrund:**

Patientenorientierung entwickelt sich politisch und gesellschaftlich zum entscheidenden Leitbild unseres Gesundheitssystems, doch in der praktischen Umsetzung zeigen sich Probleme durch konfligierende Ziele und Interessen.

**Ziel der Arbeit:**

Darstellung des Begriffs Patientenorientierung sowie möglicher Messmethoden mit besonderem Fokus auf den deutschen Sprachraum.

**Material und Methoden:**

Narrative Übersichtsarbeit durch Literatursuche in Medline, der Cochrane Library, PsyINfo und CINHAL. Aus den Ergebnissen wird das Konzept von „Patient-Reported Experience Measures“ (PREMs) und ein PREM-System entwickelt.

**Ergebnisse:**

Die Patientenorientierung ist kein abgeschlossenes theoretisches Konstrukt. Aktuell werden 16 Dimensionen der Patientenorientierung unterschieden. Die am weitesten verbreitete Messmethode zur Erfassung der Patientenorientierung sind PREMs. Anders als in anderen Ländern gibt es im Deutschen nur eine begrenzte Anzahl von PREMs, deren methodologische Messungsqualität häufig unklar ist. Wesentliche chirurgische Aspekte der Patientenversorgung werden durch vorliegende deutschsprachige PREMs nicht erfasst. Es wird ein PREM-System in deutscher Sprache entwickelt und der Forschungsbedarf aufgezeigt.

**Diskussion:**

Anders als in anderen Ländern gibt es in deutscher Sprache kein zusammenhängendes PREM-System. Die systematische Erfassung der Patientenorientierung ist daher insbesondere in der Chirurgie aktuell nicht adäquat möglich. Wesentliche methodologische, organisatorische, regulatorische und finanzielle Aspekte müssen bewältigt werden, bevor die Patientenorientierung als fundiertes Konstrukt im klinischen Alltag implementiert werden kann.

## Abstract (en)

**Background:**

Patient-centeredness has developed into the guiding principle of healthcare policy over the last decade. However, its practical implementation is hindered by numerous problems and opposing interests.

**Objective:**

To define and elucidate the term patient-centeredness, describe evidence-based measurement tools and outline recommendations for practical implementation.

**Material and methods:**

A narrative literature review was carried out in Medline, Cochrane Library, PsyINfo and CINHAL. Based on the results the concept of Patient-Reported Experience Measures (PREM) is explained and a comprehensive PREM system is developed.

**Results:**

Patient-centeredness is a not yet clearly defined theoretical construct. Patient-centeredness covers up to 16 different aspects ranging from patient involvement to the transition of care. Patient centeredness is most frequently measured via PREMs. Contrary to other countries, there are only a limited number of validated PREMs available in German and measurement qualities are frequently unclear or insufficient. No comprehensive PREM system has been developed for the German language so far. Important aspects of surgical care are not captured by currently available German language PREMs.

**Conclusion:**

Contrary to other countries no comprehensive PREM system is available in the German language. Currently available German PREMs do not enable the adequate assessment of surgical aspects of care. Important organizational, regulatory, methodological, and financial aspects must be addressed before patient-centeredness can be implemented into every clinical practice in German-speaking countries.

## Patientenorientierung in der Chirurgie

„Das Patientenwohl ist für uns entscheidender Maßstab für gesundheitspolitische Entscheidungen, die Patientenorientierung ist das Leitbild für das gesamte Gesundheitswesen“, sagte der ehemalige Bundesgesundheitsminister Jens Spahn am 07.08.2020 in einem Interview [6]. Dieser Satz findet sich wortgleich auch im Koalitionsvertrag der Bundesregierung der zu Ende gegangenen 19. Legislaturperiode [14]. Auch der Koalitionsvertrag der neuen Bundesregierung unterstreicht die Ausrichtung am Patientenwohl. Eine Stärkung der Patientenorientierung (PO) findet sich in vielen weiteren gesundheitspolitischen (Gesetzes‑)Initiativen der vergangenen Jahre wie z. B. dem Nationalen Krebsplan und dem Patientenrechtegesetz. Den Patienten „ins Zentrum unseres Handelns zu stellen“ und die PO zum wichtigen Maßstab des Handels zu machen, liest man als (Marketing‑)Slogan und Unternehmensleitbild auf den Homepages fast jeder Klinik oder jeden Unternehmens der Gesundheitsbranche.

## Probleme der Patientenorientierung in Deutschland

Dieser Wille zur Stärkung der PO durch gesetzliche Initiativen steht im Widerspruch zum bestehenden Mangel an PO auf der Mikroebene (Arzt-Patienten-Kontakt). Krankenhäuser in Deutschland sind für ihre fehlende PO bekannt: „Ärzte, Krankenhäuser und Krankenkassen müssen endlich begreifen, dass sie sich an den Patientenbedürfnissen orientieren müssen, wenn sie zukunftsfähig sein wollen“, sagte Staatssekretär Karl-Josef Laumann, ehemaliger Beauftragter der Bundesregierung für die Belange der Patientinnen und Patienten sowie Bevollmächtigter für Pflege der Bundesregierung [1]. Patienten wünschen sich mehr Zeit und Zuwendung durch die behandelnden Ärzte und Pflegenden, bemängeln fehlende Informationen und mangelnde Involvierung in Entscheidungen über ihre eigene Gesundheit [7]. Mehr als 80 % der Patienten wünschen sich eine partizipative Entscheidungsfindung, doch nur 45 % sehen diese auch realisiert [7]. In einer Untersuchung zur Patientenzentrierung medizinischer Versorgung in Deutschland gab eine Vielzahl von Befragten Mängel in der Implementierung in allen Bereichen der Patientenzentrierung an [33].

Woran liegt also diese offensichtliche Diskrepanz zwischen der Versorgungswirklichkeit und den politischen und institutionellen Willensbekundungen? Der Sachverständigenrat zur Begutachtung der Entwicklung im Gesundheitswesen hat bereits 2003 in seinem Gutachten darauf hingewiesen, dass sich der Mangel an PO durch „… Widerspruch (der Patientenorientierung) zu einer Reihe von Leitbildern, Verhaltensmustern und Organisationsabläufen bei Institutionen und Professionen des Gesundheitswesens (erklärt). Ebenso konfligieren wichtige politische Zielbündel, wie z. B. eine strikte ‚Systemorientierung‘ … als auch eine strikte ‚Kostenorientierung‘ aller Beteiligten, und gesetzliche Rahmenvorgaben, wie strenge sektorale Versorgungsgliederungen oder fallpauschalierende Vergütungen mit dem Ziel der Patientenorientierung“ [26].

## Was versteht man unter Patientenorientierung?

**Figure 1:**
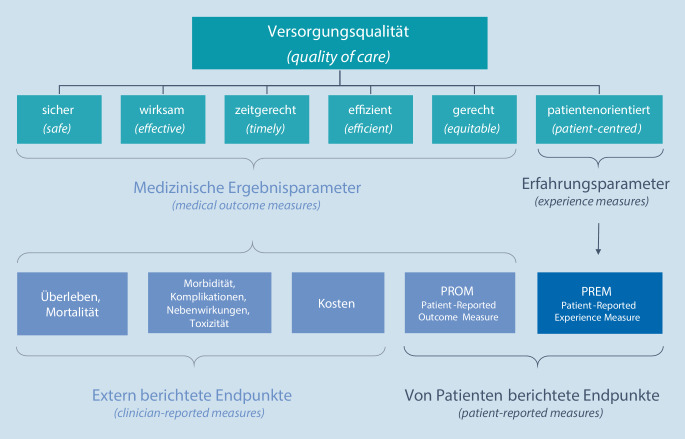

Hierdurch wird klar, dass die PO, im Englischen meist Patientenzentrierung („patient-centeredness“) genannt, nur einer von mehreren Aspekten eine hochwertigen Gesundheitsversorgung ist. Die PO wird vom Institute of Medicine (IOM) neben einer sicheren („safe“), wirksamen („effective“), zeitgerechten („timely“), effizienten („efficient“) und gerechten („equitable“) Versorgung als 6. Säule einer hochwertigen Gesundheitsversorgung („quality of care“) angesehen (Abb. 1; [12]). Diese Definition wird auch in Deutschland durch das Institut für Qualitätssicherung und Transparenz im Gesundheitswesen (IQTIG) in leicht abgewandelter Formulierung genutzt [3].

Weder das IOM noch das IQTIG definieren jedoch genauer, welche inhaltlichen Dimensionen zur PO („patient-centeredness“) gehören. Ursächlich hierfür ist die fehlende Klarheit im Konzept der PO sowie teils widersprüchliche oder überlappende Modelle [8, 17, 22, 27]. Dies spiegelt sich auch in den verschiedenen öffentlichen Äußerungen aus Politik, dem Gesundheitswesen und der Wirtschaft wider: „Während Gesundheitspolitiker und Ökonomen ‚Patientenorientierung‘ oftmals mit ‚Kundenorientierung‘ gleichsetzen und damit die Forderung einer Neuausrichtung des Gesundheitswesens verbinden, steht für Patientenvertreter eine Autonomie und Souveränität achtende Gesundheitsversorgung im Mittelpunkt. Patientenorientierung aus der Perspektive der Pflege stellt zudem den Patienten als Subjekt ins Zentrum von Betreuung und Therapie“ [21]. Letzteres dürfte auch für die Chirurgie gelten.

Scholl et al. haben 2014 durch eine systematische Übersichtsarbeit aus 417 Publikationen insgesamt 15 Dimensionen der „patient-centeredness“ identifiziert und diese zu einem integrativen Modell der PO zusammengefasst [27]. Im weit verbreitenden Picker-Modell kommt als 16. Dimension „effektive Behandlung durch vertrauenswürdiges Personal“ hinzu [4]. Tab. 1 gibt einen Überblick über Dimension der PO in gängigen Patient-centeredness-Modellen. Hierbei wird deutlich, dass sich Dimensionen der PO mit Aspekten der Versorgungsqualität (Abb. 1) überschneiden. Ein abschließendes, international anerkanntes Konzept der PO bildet sich also erst langsam heraus.

**Table 1:** 

	Scholl et al. [27]	Wong et al. [32]	Picker [4, 13]	Mead und Bower [17]
1	Die Patientin/der Patient als einzigartige Person („patient as a unique person“)	Patientenpräferenz („patient preference“)	Respekt für Patientenpräferenzen	Die Patientin/der Patient als Person („*the patient-as-person*“)
2	Biopsychosoziale Perspektive („biopsychosocial perspective“)	–	–	Biopsychosoziale Perspektive („biopsychosocial perspective“)
3	Essenzielle Eigenschaften der Behandler („essential characteristics of the clinician“)	–	–	Die Ärztin/der Arzt als Person („*the doctor-as-person*“)
4	Einbindung von Patienten in ihre Behandlung („patient involvement in care“)	Patientenpräferenz („patient preference“)	Beteiligung an Entscheidungen	Teilen von Macht und Verantwortung („sharing power and responsibility“)
5	Einbindung von Angehörigen und Freunden („involvement of family and friends“)	Familie und Freunde („family and friends“)	Einbeziehung der Familie und Freunde	Therapeutische Allianz („therapeutic alliance“)
6	Physische Unterstützung („physical support“)	Physische Unterstützung („physical support“)	Physisches Wohlbefinden, saubere und sichere Umgebung	–
7	Emotionale Unterstützung („emotional support“)	Emotionale Unterstützung („emotional support“)	Emotionale Unterstützung, Empathie, Respekt	–
8	Behandler-Patienten-Kommunikation („clinician-patient communication“)	Information und Unterweisung („information and education“)	Klare, verständliche Information und Unterstützung für die Selbstversorgung	Teilen von Macht und Verantwortung („sharing power and responsibility“)
9	Stärkung von Selbstbestimmung u. Autonomie („patient empowerment“)	–		
10	Patientinnen- u. Patienteninformation („patient information“)	–		Therapeutische Allianz („therapeutic alliance“)
11	Zugang zur Versorgung („access to care“)	Zugang zur Versorgung („access to care“)	Schneller Zugang zu zuverlässiger Gesundheitsversorgung	–
12	Integration medizinischer u. nichtmedizinischer Behandlung („integration of medical and non-medical care“)	–	–	–
13	Koordination und sektorenübergreifende Versorgung („coordination and continuity of care“)	Koordination der Versorgung („coordination of care“)	Kontinuität der Behandlung und geregelte Überleitsysteme	–
		Kontinuität der Versorgung („continuity and transition of care“)		
14	Teamwork („teamwork and teambuilding“)	–	–	Therapeutische Allianz („therapeutic alliance“)
15	Behandler-Patienten-Beziehung („clinician-patient relationship“)	–	–	
16	–	–	Effektive Behandlung durch vertrauenswürdiges Fachpersonal	

Verschiedene Modelle betonen unterschiedliche Dimensionen der „patient-centeredness“. Scholl et al. aus [27]; Picker aus [4, 13], Wong et al. aus [32], Mead und Bower aus [17]. Falls es sich um englischsprachige Originalpublikationen handelt, stehen die englischen Begriffe in Klammern, um Missverständnisse zu vermeiden

## Wie misst man Patientenorientierung?

Verschiedene Methoden sind für die Messung der PO beschrieben worden, u. a. Fokusgruppen, Interviews, öffentliche Treffen, „comment cards“, Beschwerdemanagementsysteme oder Fragebögen [28]. Einen „Goldstandard“ gibt es nicht und kann es, wie weiter unten ausgeführt, auch nicht geben. Die Erfassung der PO mittels validierter Fragebögen hat sich international durchgesetzt, da sie höchste Generalisierbarkeit und Standardisierung versprechen. Diese Umfragenwerkzeuge werden gemeinhin „Patient-Reported Experience Measures“ (PREMs) genannt. Der Name benennt schon einen wichtigen Aspekt von PREMs: Die PO soll anhand der Erfahrung der Patienten in einem spezifischen Gesundheitskontext erfasst werden.

Während die Messung der ersten fünf Säulen des IOM „Quality-of-Care“-Modells mittels klinischer Endpunkte erfolgt, wird die PO also mittels „experience measures“ erfasst. Aller Erfahrung nach bereitet die Bewertung der ersten fünf IOM-Säulen durch klinische Endpunkte den meisten Chirurgen weit weniger Probleme als die Beurteilung der PO.

PREMs dienen nach Bull et al. drei Zielen [5]:

1. PREMs ermöglichen es Patienten, über ihre persönliche Versorgungserfahrung zu reflektieren.
2. PREMs dienen einzelnen Kliniken oder Abteilungen als Qualitätssicherungsinstrument und ermöglichen gezielte Verbesserungen.
3. PREMs erlauben eine Messung der Patientenorientierung und Versorgungsqualität über Kliniken hinweg und ermöglichen damit das Benchmarking.

Unzählige Publikationen beschäftigen sich mit der Frage, wie die PO, gemessen mittels PREMs, und klinische Ergebnisparameter zusammenhängen. Die Frage wird kontrovers diskutiert, insbesondere in Ländern wie den USA, in denen PREM-Bewertungen Auswirkungen auf die Bezahlung von Kliniken haben („pay-for-performance“). Diese Diskussion erscheint jedoch müßig, denn, wie oben ausgeführt, erfassen PREMs einen dezidiert anderen Aspekt der Versorgungsqualität als klinische Ergebnisparameter (Abb. 1; [16]).

## PREMs, PROMs, Zufriedenheit

PREMs dienen also der Erfassung der *Erfahrung* des Patienten in einem bestimmten Versorgungskontext (z. B. in einer chirurgischen Abteilung). Die „patient experience“ unterscheidet sich daher grundlegend von der Zufriedenheit („satisfaction“). Erstens wird Zufriedenheit global bewertet, während PREMs sehr detailliert einzelne Erfahrungen abfragen. Als Beispiel eine Frage aus der deutschen Version des U. S.-amerikanischen HCAHPS-Fragebogens: „Wie oft haben die Ärzte Sie während dieses Krankenhausaufenthaltes höflich und mit Respekt behandelt? Nie/Manchmal/Meistens/Immer“ [30]. Zweitens ist die Zufriedenheit von vielen Einflussfaktoren („confounder“) wie z. B. der Erwartungshaltung von Patienten abhängig, die eine generalisierbare und valide Auswertung erschweren [23]. Fragen zur Patientenerfahrung hingegen können zur Konstruktion valider und reliabler Fragebögen genutzt werden [16]. In der Literatur und auch bei Fragebögen werden die Begriffe PO und Zufriedenheit („satisfaction“) jedoch nicht immer klar voneinander getrennt. Eine genaue Analyse des Fragebogens ist also in jedem Einzelfall notwendig.

Bei PREMs handelt es sich um eine Messmethode, bei der Patienten eigenständig Fragebögen ausfüllen, um ihre „patient-experience“ zu bewerten. PREMs sind insofern mit „patient-reported outcome measures“ (PROMs) verwandt (Abb. 1). Ähnlich wie PREMS werden auch PROMs definiert als „… any outcome evaluated directly by the patient himself or herself and is based on patient’s perception …“ [9]. Anders als PREMs bezieht sich die „patient’s perception“ bei PROMs jedoch auf klinische Ergebnisparameter der Erkrankung oder der Behandlung (z. B. Schmerzen, Abgeschlagenheit oder die Lebensqualität) und nicht auf die „patient experience“ zur Messung der PO (Abb. 1). Es gibt noch einen weiteren wesentlichen Unterschied zwischen PREMs und PROMs. Wie oben dargelegt (Tab. 1), setzt sich die PO aus verschiedenen Dimensionen zusammen. PREMs sind somit immer multidimensional. PROMs hingegen können sowohl eindimensional sein (z. B. die Messung des Schmerzes mittels der Numeric Rating Scale) als auch multidimensional (z. B. die Ermittlung der Lebensqualität mittels eines multidimensionalen Fragebogens). Zum Teil sind die Grenzen zwischen PREMs und PROMs aber fließend.

## PREM-Kategorien

Ein wichtiger Aspekt der PREMs ist, dass diese nicht per se universell einsetzbar sind, sondern jeweils bestimmte, patientenrelevante Aspekte der Versorgung abfragen. Dies erklärt, dass eine Vielzahl von PREMs je nach Kontext entwickelt wurde. Wir schlagen eine grobe Kategorisierung von PREMs in vier Gruppen vor:

1. *Generische PREMs* erfassen allgemeine Aspekte der PO und sind somit in verschiedenen Versorgungskontexten, Fachgebieten, Erkrankungen und Ländern einsetzbar.
2. *Fachspezifische PREMs *erfassen Besonderheiten in der „patient experience“ eines bestimmten Fachs. So würde z. B. der Aspekt der Schmerzfreiheit in allen chirurgischen Fächern ein wesentlicher Aspekt der Versorgung darstellen, aber in internistischen Fächern von weniger großer Bedeutung sein.
3. *Behandlungspfadspezifische PREMs *(„healthcare pathway-specific“) dienen dazu die PO eines bestimmten Behandlungspfads über Fächer- und Sektorengrenzen hinweg zu erfassen, z. B. für Tumorpatienten.
4. *Krankheitsspezifische PREMs *dienen dazu die PO spezifischer Patientengruppen zu erfassen.

**Figure 2:**
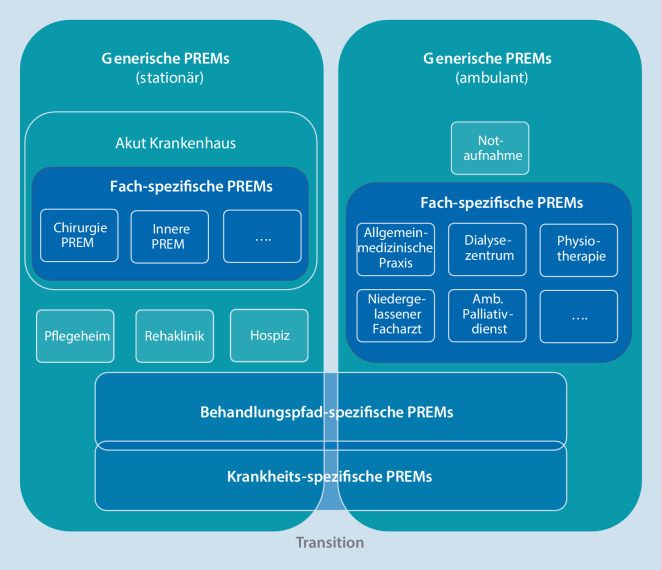

Die verschiedenen Kategorien zeigen Überlappungen. In Abb. 2 wird der Versuch unternommen, die verschiedenen PREM-Kategorien schematisch zu einem zusammenfassenden PREM-System anzuordnen.

Je nach Versorgungskontext, Gesundheitssystem, kulturellem Hintergrund oder Erkrankung müssen PREMs teils unterschiedliche Fragen enthalten, um spezielle Aspekte der PO und der Versorgungsqualität zu erfassen. Aus dem Gesagten ergibt sich auch, dass in dem Maße, wie sich das Gesundheitssystem oder die geplante Ausrichtung der Versorgung ändern, auch neue PREMs oder zusätzliche PREM-Fragen notwendig sind. Somit kann es den abschließenden „Goldstandard“ bei PREMs nicht geben. Als Beispiel sei die sektorenübergreifende Versorgung genannt, die in Deutschland als mangelhaft gilt. Um diese zu erfassen, bedarf es anderer PREM-Fragen, als sie in einem rein stationären PREM abgebildet sind [20].

## Messen PREMs denn wirklich die Patientenorientierung?

Inwieweit ein PREM sein erklärtes Ziel, nämlich die PO über die Messung der „patient experience“ in einer bestimmten Versorgungssituation zu erfassen, erreicht, hängt davon ab, wie methodologisch aussagekräftig die verwendeten Messmethoden sind. Die Messungen der PO mittels nicht validierter PREM-Instrumente ist nicht zielführend und stellt eine Verschwendung von Ressourcen dar. Leider ist diese Praxis weit verbreitet [24]. Die Güte von PROMs oder PREMs lässt sich anhand psychometrischer Gütekriterien messen. Diese Gütekriterien („measurement properties“) sind Reliabilität, Validität, Responsivität, Interpretierbarkeit, Machbarkeit, Angemessenheit und Akzeptanz [10, 19]. Leider werden die Definitionen sowie die Taxonomie dieser Gütekriterien nicht einheitlich verwendet, was zu erheblicher Verwirrung sorgen kann. Die COSMIN-Gruppe (COnsensus-based Standards for the selection of health Measurement INstruments) hat in den letzten Jahren Leitlinien zur Entwicklung und Bewertung patientenberichteter Messmethoden (PREMs, PROMs) publiziert, die sich international durchgesetzt haben [19, 25] und die sich auf der COSMIN-Homepage finden lassen (https://www.cosmin.nl/).

## Internationale PREM-Systeme

PREMs spielen in vielen Ländern eine große Bedeutung in der Bewertung, Bezahlung und Organisation des jeweiligen Gesundheitssystems. In Schweden wurde eines der ersten PREMs entwickelt und auch landesweit eingesetzt: der Quality of Care from the Patients’ Perspective (QPP; [15]) sowie dessen Kurzversion (QPPS; [31]) sind feste Bestandteile des schwedischen Gesundheitssystems. Manche Länder sind jedoch noch einen Schritt weiter gegangen und haben generische, fachspezifische, behandlungspfadspezifische und krankheitsspezifische PREMs zu einem umfassenden PREM-System verknüpft. PREM-Systeme finden sich u. a. in den USA (CAHPS; https://www.ahrq.gov/cahps/index.html), England (NHS), den Niederlanden (Consumer Quality Index, CQI) und Norwegen (Norwegian Knowledge Centre for the Health Services). Auch zwei Organisationen haben zusammenhängende PREM-Fragebögen entwickelt: das Picker Institute sowie, in begrenzten Umfang, die Quality-of-Life-Gruppe der European Organisation for Research and Treatment of Cancer (EORTC) für den Bereich der Krebsbehandlung. Die Ergebnisse der U. S.-amerikanischen generischen In-hospital-PREMs (HCAHPS) dienen dem Vergleich zwischen verschiedenen Kliniken. Auch auf die Bezahlung haben die Ergebnisse Einfluss („pay-for-performance“). Basierend auf den Ergebnissen wurden in der Folge ganze Kliniken und Abteilungen umstrukturiert [18]. Eine Übersicht der PREM-Systeme findet sich in Tab. 2.

**Table 2:** 

	Generische PREMs	Fachspezifische PREMs	Behandlungspfadspezifische PREMs	Krankheitsspezifische PREMs
	Provider-spezifisch			
USA (CHAPS)	Hospital Survey	CAHPS Surgical Care Survey	CAHPS Cancer Care (CC-CAHPS)	CAHPS Dental Plan
	(HCAHPS)	CAHPS Outpatient and Ambulatory Surgery Survey (OAS CAHPS)		CAHPS Experience of Care and Health Outcomes in Mental Health (ECHO)
	Clinician and Group Survey (CG-CAHPS)	CAHPS In-Center Hemodialysis Survey		
	CAHPS Home and Community-Based Services Survey (HCBS CAHPS)			
	CAHPS			
	Emergency Department Survey (ED CAHPS)			
	CAHPS Home Health Care Survey			
	CAHPS Hospice Survey			
	CAHPS Nursing Home Surveys			
England (NHS)	NHS Inpatient Survey (via Picker)	NHS Maternity Survey	–	NHS Inpatient Mental Health Survey (via Picker)
	NHS Outpatient Survey (via Picker)			NHS Community Mental Health (via Picker)
	NHS Children and Young People Patient Experience Survey (via Picker)			
	NHS Urgent and Emergency Care Survey (Via Picker)			
Niederlande (CQI)	CQI Inpatient Hospital Care	CQI Cataract Surgery	CQI Cancer Care	CQI for Chronic Dialyses
	CQI General Practitioner	CQI Palliative Care		CQI Diabetes
	CQI Care for the Elderly („nursing home“)	CQI Social Care		CQI Asthma
	CQI for the Accident and Emergency Department (CQI A&E)			CQI Heart Failure
Norwegen (NKCHS)	Hospital Outpatients (OPEQ)	Next-of-Kin of Pediatric Inpatients	–	Psychiatric Inpatients (PIPEQ)
	Hospital Inpatients (PEQ)			Psychiatric Outpatients (POPEQ)
Picker-Institut	Picker Patient Experience Questionnaires (PPE-40, PPE-15)	Picker Primary Care Survey (Allgemeinmedizin)	Picker Cancer Survey	Coronary Heart Disease In-Patient Experience Questionnaire
	Picker Adults Outpatient Questionnaire			Picker STROKE QUESTIONNAIRE
	Picker Adult Emergency Questionnaire			Picker Mental Health Care Questionnaire
	Picker Young Patient Survey			Picker Diabetes Questionnaire
	Picker Community Service Questionnaire			Child and Adolescent Mental Health Services Questionnaire
EORTC	–	–	IN-PATSAT32	–
			PATSAT-C33	
			OUT-PATSAT7	

*CAHPS* Consumer Assessment of Healthcare Providers and Systems, *CQI* Consumer Quality Index, *NKCHS* Norwegian Knowledge Centre for the Health Services, *EORTC* European Organisation for Research and Treatment of Cancer

## Deutschsprachige PREMs

Eine systematische Übersichtsarbeit deutschsprachiger PREMs und eine Bewertung ihrer psychometrischen Gütekriterien liegen nicht vor, wird aber von den Autoren aktuell vorbereitet. Zu den deutschsprachigen PREMs gehören z. B. die deutsche Version des amerikanischen HCAHPS-Fragebogens [30], der Picker Patient Experience Questionnaire 15 (PPE-15; [13]), die Kurzversion des schwedischen Quality from the Patients’ Perspective (QPPS; [29]), der deutsche Patient Experience Questionnaire (PEQ; [11]) sowie des Patient Experience Across Healthcare Settings (PEACS; [20]). Die deutschsprachigen PREMs stellen entweder Übersetzungen aus anderen Sprachen dar, wie der PPE-15 [13], HCAHPS [30] und QPPS [29], oder es handelt sich um PREMs, die direkt in Deutsch entwickelt wurden wie der Patient Experience Questionnaire (PEQ [11] oder PEACS [20]). Ein zusammenhängendes PREM-System, wie es andere Länder aufgebaut haben, gibt es im deutschen Sprachraum nicht. Chirurgiespezifische PREMs liegen in deutscher Sprache, anders als im englischen Sprachraum, nicht vor.

## Was ist konkret zu tun?

Die Ausrichtung an der PO entspricht einem politischen und gesellschaftlichen Wandel, dem wir uns als Chirurgen stellen müssen. Damit die Dimensionen der PO (Abb. 1) im ärztlichen Alltag genutzt werden können, erscheinen uns folgende Entwicklungen notwendig:

1. *Deutschsprachige PREMs.* Für wesentliche Bereiche der Gesundheitsversorgung liegen in deutscher Sprache keine validen PREMs vor (z. B. Chirurgie). Solche PREMs sollten dringend entwickelt und validiert werden.
2. *Digitalisierung.* Die routinemäßige Erfassung von PREMs benötigt datenschutzkonforme digitale Systeme (Apps, Onlinefragebögen). Auch wenn einzelne Anbieter solche Systeme für die Erfassung von PROMs bereitstellen, ist die Integration in bestehende KIS und Praxissoftware nicht ausgerollt und stellt ein dringendes Handlungsfeld dar.
3. *PREM-basierte Interventionen.* Welche Rückschlüsse und Verbesserungen lassen sich konkret aus den Ergebnissen einzelner PREM-(Sub‑)Skalen für die Kliniken, Abteilungen und Praxen ableiten? Gibt es standardisierbare „Interventionen“, die einzelne Dimensionen der PO verbessern? Oder sind jeweils spezifische organisatorische und strukturelle Maßnahmen notwendig? Zu all diesen Fragen fehlen qualitativ hochwertige Studien.
4. *Holistische Betrachtungsweise.* Auch wenn die PO ein wesentlicher Aspekt der Versorgung darstellt, sollten, wie in Abb. 1 dargestellt, medizinische Ergebnisparameter, PROMs und PREMs zusammen betrachtet werden, um ein ganzheitliches Bild der Versorgungsqualität zu erhalten. Es ist also notwendig, PREMs zusammen mit der standardisierten Erfassung von PROMs und validierten, patientenrelevanten medizinischen Endpunkten auszuwerten und darzustellen. Hierzu wäre die Entwicklung von „standard outcome sets“ für spezifische Patientengruppen notwendig. Diesbezüglich sei u. a. auf die COMET-Initiative (www.comet-initiative.org) und ICHOM (www.ichom.org) verwiesen.

Die PO zielt aber auch auf eine viel größere Fragestellung ab: Wie sollten Gesundheitsinstitutionen in der Zukunft organisiert sein, um die PO zu gewährleisten? Im Ausland ist zu beobachten, wie die Orientierung an PREMs zu erheblichen Umstrukturierungen mit Orientierung am Patienten [18] oder Fokussierung auf krankheitsorientierte Einheiten mit hoher organisatorischer Autonomie führt [2]. Hochspezialisierte Leistungen können in solchen Systemen auch intersektoral mit hoher Qualität und PO erbracht werden, ohne die in Deutschland zu beobachtenden Organisationszentralisierung, die häufig zu einer Depersonalisierung der Medizin führt. Dies bedeutet aber ein klares Umdenken, weg von einer immer noch stark hierarchisierten Klinikstruktur hin zu einem kollegialen Organisationsmodell mit klar definierten Zuständigkeiten. Managementaufgaben können in einem solchen System auf mehrere Schultern auch interdisziplinär verteilt werden. Klare medizinische Zuständig- und Eigenverantwortlichkeiten führen dann auch zu einer verbesserten intersektoralen Zusammenarbeit auf Augenhöhe.
